# Emergence of Arboviruses in the United States: The Boom and Bust of Funding, Innovation, and Capacity

**DOI:** 10.3390/tropicalmed5020096

**Published:** 2020-06-06

**Authors:** Rebekah C. Kading, Lee W. Cohnstaedt, Ken Fall, Gabriel L. Hamer

**Affiliations:** 1 Department of Microbiology, Colorado State University, Immunology and Pathology, Fort Collins, Colorado, CO 80523, USA; 2 United States Department of Agriculture, Arthropod-borne Animal Diseases Research Unit, Agricultural Research Service, Manhattan, Kansas, KS 66502, USA; lee.cohnstaedt@usda.gov; 3 BioQuip Products, Rancho Dominguez, California, CA 90220, USA; ken@bioquip.com; 4 Department of Entomology, College of Agriculture and Life Sciences, Texas A&M University, College Station, Texas, TX 77843, USA; ghamer@tamu.edu

**Keywords:** mosquito, emerging virus, outbreak, surveillance, trap, *Aedes*, *Culex*, Zika virus, West Nile virus

## Abstract

Mosquito-borne viruses will continue to emerge and generate a significant public health burden around the globe. Here, we provide a longitudinal perspective on how the emergence of mosquito-borne viruses in the Americas has triggered reactionary funding by sponsored agencies, stimulating a number of publications, innovative development of traps, and augmented capacity. We discuss the return on investment (ROI) from the oscillation in federal funding that influences demand for surveillance and control traps and leads to innovation and research productivity.

## 1. Introduction

While outbreaks of infectious diseases are devastating to human and animal populations, surges in private and public funding for outbreak pathogens have positively influenced research and innovation. Contemporary outbreak response efforts now incorporate digital media; electronic surveillance tools; mathematical modelling; sequencing; and teams of experts that include anthropologists, social scientists, and other diverse disciplinarians [1]. Outbreak stimulus funding leads to increased scientific productivity on outbreak pathogens, and incites innovation [2]. For example, an innovation workshop convened after an anthrax bioterrorism attack in 2001, to discuss new surveillance and pathogen detection approaches [3]. The Ebola Grand Challenge program, funded by the US Centers for Disease Control and Prevention (CDC), United States Agency for International Development (USAID), the United States Executive Office, and the Department of Defense, provided financial backing to 14 innovative projects to improve the response to Ebola outbreaks. These projects included protective suits, health care and technology solutions, and decontaminants [4]. The USAID Zika Grand Challenge program in 2016 received over 900 applications and provided 30 million dollars to innovative ideas to address Zika virus transmission [5]. Currently, during the COVID-19 pandemic, innovations are emerging such as new ventilators, drones delivering medical supplies, and the use of artificial intelligence in medicine [6,7,8,9]. Ideally, these scientific advances will aid in combatting the current epidemic as well as fuel innovation in preparedness for future emerging disease events, leading to return on investment (ROI).

The introduction and spread of numerous mosquito-borne viruses around the globe has also shepherded a wave of innovation to improve upon industry-standard sampling techniques that more efficiently capture the target vector species, physiological cohort, or virus-infected population of vectors [10]. Ramirez et al. [10] recently reviewed traditional surveillance approaches in the context of novel innovations that have advanced surveillance capacity, including pathogen surveillance from sugar feeding vectors, next-generation sequencing (eDNA (environmental DNA) for presence or absence of vectors in habitats), and xenosurveillance for vectors and pathogens. Additionally, modernized sampling strategies have also been expanded to incorporate technical advancements in such areas as infrared scanning, citizen science, and drones [11,12,13,14]. Driving the introduction and incorporation of novel trapping approaches to surveillance programs is the persistent emergence of mosquito-borne pathogens. 

## 2. Arbovirus Emergence in the U.S.

Many arboviruses cause a significant disease burden to the public each year. West Nile virus (WNV) (introduced in 1999), St. Louis encephalitis virus (SLEV), Jamestown Canyon virus (JCV), La Crosse encephalitis virus (LACV), Eastern equine encephalitis virus (EEEV), and Powassan (POWV, tick-borne) are tracked through the CDC database, ArboNET [15]. In the last several years, incidence of JCV and POWV has been increasing [15,16,17,18]. Additionally, a multistate outbreak of EEEV occurred in the northeastern US in 2019, in which the 34 reported cases far exceeded the historical average of around eight cases/year [19]. Through the first half of the 20th century, Western equine encephalitis virus (WEEV) was also widespread and caused significant morbidity and mortality in humans and horses; however, this virus has largely disappeared from most areas over the past several decades for unconfirmed reasons, with the last human case in the United States reported in 1999 [20,21]. 

In 2013, chikungunya (CHIKV) was first reported in the Americas, followed by Zika virus (ZIKV) two years later [22,23]. These virus invasions came during a time of increasing dengue virus (DENV) incidence, range expansion, and replacement of dominant serotypes in different geographic areas [24,25,26]. The Pan American Health Organization (PAHO) reported a 30% increase in the number of dengue cases, from 7,641,334 between 2001 and 2010 to 10,851,043 between 2011 and 2017 [26,27]. Intensive transmission of *Aedes*-borne viruses in the Americas coupled with the expanding range of *Ae. aegypti* mosquitoes presents a continual threat of these viruses gaining a foothold in the United States as well [28,29,30]. To date, cases of DENV, ZIKV, and CHIKV in the United States have predominately been traveler-associated, with the exception of local transmission of ZIKV in Florida and Texas during 2017 [31,32].

## 3. Federal Funding and Publications in Response to Arboviral Emergence

**Figure 1:**
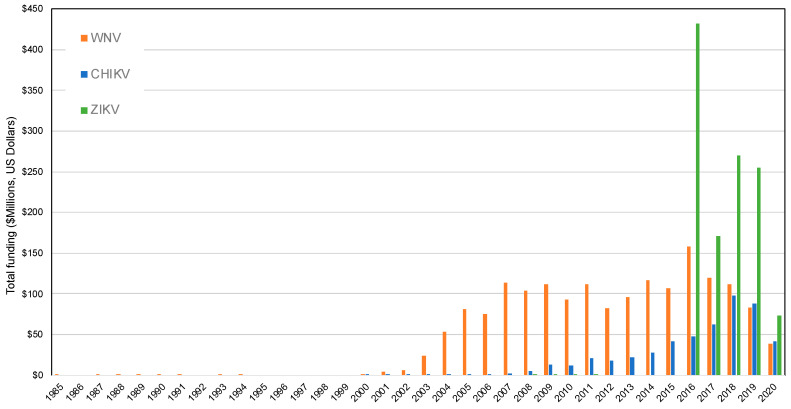
Total funding by the National Institutes of Health based on the National Institutes of Health (NIH) Reporter search query of projects matching West Nile virus (WNV), chikungunya virus (CHIKV), and Zika virus (ZIKV), from 1985 to 2020. Expenditures in 2020 are incomplete as search was conducted on 16 May 2020. Graphed values were corrected for inflation by the Consumer Price Index referenced to 2019 provided by the U.S. Bureau of Labor Statistics.

**Figure 2:**
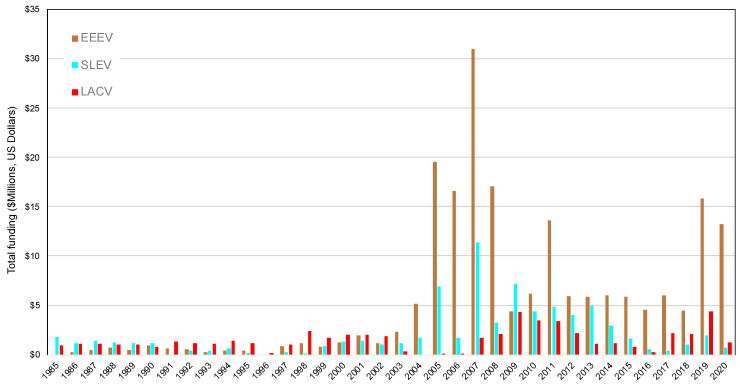
Total funding by the National Institutes of Health based on the NIH Reporter search query of projects matching Eastern equine encephalitis virus (EEEV), St Louis encephalitis virus (SLEV), and La Crosse encephalitis virus (LACV) from 1985 to 2020. Expenditures in 2020 are incomplete as search was conducted on 16 May 2020. Graphed values were corrected for inflation by the Consumer Price Index referenced to 2019 provided by the US Bureau of Labor Statistics.

When a mosquito-borne virus rapidly spreads around the world and becomes a public health emergency of international concern, federal agencies allocate substantial funds to fight the virus. In the U.S., Congress approves these funding packages, such as the $1.1 billion for Zika virus in September, 2016 [33]. This stimulus funding provides agencies such as the National Institutes of Health (NIH), the Centers for Disease Control and Prevention (CDC), and the National Science Foundation the resources to allocate funds to public health agencies and researchers to enhance outbreak surveillance and response efforts. As a proxy for this federal spending allocated to different emerging arboviruses, we used the NIH RePORTER search query to estimate the funding of projects related to the emergence of WNV, CHIKV, and ZIKV. We used the key words of ‘’West Nile virus’’, ‘’chikungunya virus’’, and ‘’Zika virus’’ in the search feature which queries all project titles, abstracts, and scientific terms. To compare these invasive mosquito-borne viruses to those endemic to the United States, we also included three endemic mosquito-borne viruses that have resulted in continual transmission, including human disease, in the last several decades. These additional virus searches were performed for ‘’Eastern equine encephalitis virus’’, ‘’St Louis encephalitis virus’’, and ‘’La Crosse encephalitis virus’’. The search for St. Louis encephalitis virus returned more projects with ‘’St’’ than with ‘’Saint’’. The search was performed on 16 May 2020, and we included all years from 1985–2020 in each search. NIH-funded projects have a record for each fiscal year of a project (e.g., a 5-year NIH R01 has five records). The projects include international and national investigators and project locations. The search for WNV resulted in 2803 records for a total of $1,493,811,562 in funding; CHIKV matched 960 records for $477,117,414 in funding; and ZIKV matched 1332 records for $1,164,293,217 in funding (Figure 1). The search for EEEV matched 310 records for a total of $165,906,376 in funding; SLEV matched 143 records for $59,372,038 in funding; and LACV matched 126 records for $41,122,419 in funding (Figure 2). The rise in NIH-funded projects for the invasive arboviruses rose quickly following their introduction into the U.S., especially for ZIKV. The NIH-funding for endemic arboviruses was steadier over the 35 time year period, especially for LACV.

In response to WNV and ZIKV, the CDC supports detection and response capacity to local agencies by providing states Epidemiology and Laboratory Capacity (ELC) cooperative agreements with all 50 states and US territories. For the first five years following the introduction of WNV into the U.S., the CDC issued ELC funds which were reduced in 2004. A survey by the Council of State and Territorial Epidemiologists in 2005 showed several enhancements to surveillance, laboratory, and control capacity that were made possible as the result of ELC funding [34]. However, after yearly reductions in ELC funds, a survey in 2013 revealed many of these programs had reduced or lost surveillance and response measures [35]. Despite this lack of sustained funding and reduced infrastructure, WNV continues to have periodic large epidemics [36]. The advent of CHIKV and ZIKV to the Americas caught many U.S local, state, and federal agencies off guard with a lack of knowledge of, lack of surveillance of, or inability to control *Aedes* (*Stegomyia*) mosquitoes. Once again, along with the NIH who spent over $400 million in 2016 on ZIKV-related projects, the CDC administered $97 million in supplementary Zika ELC stimulus funds to states to help programs re-bound and shift from a *Culex*-centric focus to *Aedes* [37]. This pulse in funding helped improve the surveillance and control of *Aedes* mosquitoes, although the fading of Zika virus in the national news and the reduction of these ELC funds [38] has resulted in many programs again reducing their capacity.

**Figure 3:**
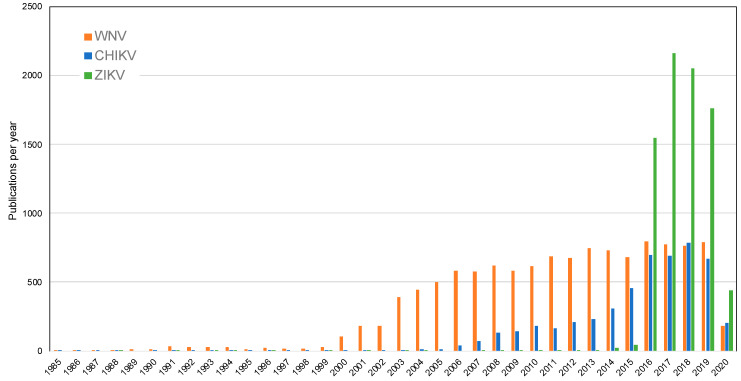
Results of a Basic Search of the Web of Science Core Collection for West Nile virus (WNV), chikungunya virus (CHIKV), and Zika virus (ZIKV), from 1985 to 2020. Publications in 2020 are incomplete as search was conducted on May 16, 2020.

**Figure 4:**
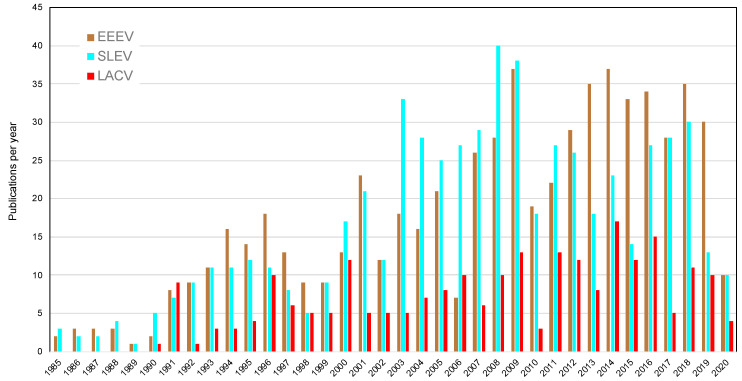
Results of a Basic Search of the Web of Science Core Collection for eastern equine encephalitis virus (EEEV), St. Louis encephalitis virus (SLEV), and La Crosse encephalitis virus (LACV) from 1985 to 2020. Publications in 2020 are incomplete as search was conducted on May 16, 2020.

Along with the pulse in funding and attention by media and the public, surges in peer-reviewed publications occur following the emergences of arboviruses. To quantify these publications, we performed a “Basic Search” of the Web of Science Core Collection for the key words of “West Nile virus”, “chikungunya virus”, and “Zika virus”. As with the NIH RePORTER search, we included “Eastern equine encephalitis virus”, “St Louis encephalitis virus”, and “La Crosse encephalitis virus”. The search for St. Louis encephalitis virus returned more publications with “St” than with “Saint”. The year range for the search was 1985 to 2020, and the search was conducted on May 16, 2020. The results yielded 11,868 records for WNV; 5088 for CHIKV; 8074 for ZIKV; 634 for EEEV; 604 for SLEV; and 238 for LACV (Figure 3 and Figure 4). The yearly pattern in publications for these three arboviruses closely matches the total funding by NIH, although publications reported on Web of Science include publications by international authors funded by different international sponsors. Publications including the endemic arboviruses as keyword searches have slowly increased over the last 35 years, but the scale of the increase in publications for invasive arboviruses is much greater.

## 4. Innovation of Mosquito Surveillance Tools

**Figure 5:**
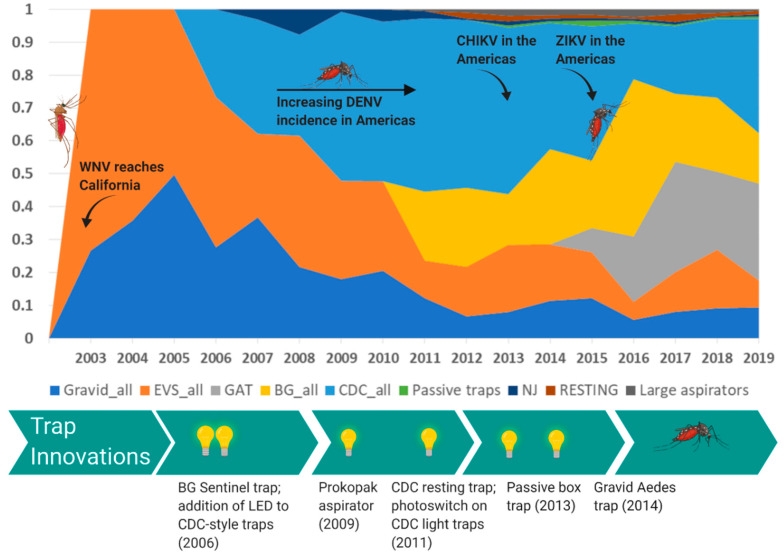
Proportion of traps sold per year by BioQuip Products. Product numbers represented included: Gravid traps (2800, 2800S), BG traps (2880, 2883), NJ light trap (2856), EVS traps (2801A, 2780, 2780NS1, 2780NS2), Centers for Disease Control and Prevention (CDC) traps (2848, 2770, 2836BQ, 2836BQX), resting traps (2799), Gravid Aedes Traps (2797), passive traps (2887, 2887P), large aspirators (2888A, 2846). Inset: Proportion of trap types sold per year by BioQuip Products. Product numbers represented included: Gravid traps (2800, 2800S), BG traps (2880, 2883), NJ light trap (2856, 2857, 2858), EVS traps (2801A, 2780, 2780NS1, 2780NS2), CDC traps (2848, 2770, 2836BQ, 2836BQX), resting traps (2799), Gravid Aedes Traps (2797), passive traps (2887, 2887P), and large aspirators (2888A, 2846).

The evolution of mosquito trapping tools targeting *Culex* and *Aedes* mosquitoes has followed suit, being driven by the emergence of these viruses, the resulting resources generated by federal agencies, and consumer demand. To visualize trends in trap usage over time, we evaluated BioQuip Products sales data in each main trap category between 2003 and 2019 (Figure 5). As the United States has experienced emergences and threats of first *Culex* and now also *Aedes*-borne viruses, innovations to traditional mosquito trapping tools targeting these vector groups have arisen, with the proportion of sales in different categories fluctuating over time in response to arbovirus outbreak and funding availability (Figure 1). The diversity of traps sold by BioQuip Products also increased substantially from two categories in 2003 to nine categories by 2014 (Figure 3) as new innovations came to market. Undoubtedly, the surge of *Aedes*-borne viruses in particular during this time period has influenced the development and sales of novel traps targeting *Ae. aegypti* mosquitoes.

### 4.1. Culex-Borne Virus Surveillance

The CDC light trap has become an industry standard for collection of mosquitoes for the purposes of arbovirus surveillance. The similar EVS (Encephalitis Vector Survey) trap [39] is also routinely used for arbovirus surveillance and operates on a similar principle as the CDC light trap using light and carbon dioxide (CO_2_) as attractants. Between 2003 and 2010, trap sales were dominated by suction traps using light and CO_2_ as attractants, and gravid traps, largely for *Culex* vectors of WNV and SLEV. Due to the sustained threat of WNV year after year [36], traps targeting *Culex* vectors are expected to remain a mainstay in the industry. 

Several specific innovations have either improved the functionality of the standard light trap or targeted different physiological cohorts of mosquitoes. In 2006, BioQuip released the first commercially available light-emitting-diode (LED) CDC-style light trap with customizable colors (Figure 3). Bayonet mounted LED chips were also available to replace the white incandescent lights in existing CDC style light traps. The passive box trap offered an alternative to CDC light traps, by providing a CO_2_-baited trap that was not reliant on a power source and could be used to sample arbovirus vectors in more remote areas [40]. A collapsible passive trap was also developed to address portability issues [41]. A photo switch option also added to light traps, allowing researchers the flexibility to set traps when convenient and conserve battery power. Finally, the CDC resting trap was introduced in 2011 for the purpose of collecting blood-engorged mosquitoes to determine vertebrate host utilization [42]. Passive and resting traps represent a smaller proportion of sales over the last several years, suggesting their utility may be serving the research community as opposed to being integrated into large-scale operational surveillance activities.

### 4.2. Aedes-Borne Virus Surveillance

Surveillance for viruses transmitted by *Ae. aegypti* mosquitoes requires very different tools and strategies than for those used for *Culex*-borne viruses. These urbanized, day-biting, container-breeding mosquitoes are generally not attracted to the suction and gravid traps that are so efficient at catching *Culex* mosquitoes [43]. The growing need for traps that effectively collected *Aedes* mosquitoes led to the innovation of the BG Sentinel Mosquito Trap in 2006 (Biogents AG, Regensburg, Germany) [44], among others. BG sentinel traps began selling at BioQuip in 2011, and sales comprised approximately 50% of the total traps sold by BioQuip in 2016 following the introduction of CHIKV and ZIKV to the Americas (Figure 3). Similarly, gravid *Aedes* traps (GAT) [45,46] were introduced between 2013 and 2014. Conceptually based on traditional gravid traps [47], these traps do not require electricity to function. GAT sales quickly grew to approximately 30% of the traps sold by BioQuip in 2016 (Figure 5). The InsectaZooka© and the Prokopak aspirator [48] are lightweight, portable field aspirators developed as an alternative to backpack models. These devices facilitate the collection of resting and engorged mosquitoes from indoor and outdoor resting sites. 

## 5. Conclusions

The emergence of mosquito-borne viruses has occurred repeatedly in recent decades, and studies predict that these emergences will continue. Much of our response to these events is reactionary, triggered by the increase in attention, funding, publication, innovation, and preventive measures for public health. The long-term impact or return on investment of outbreak spending is evidenced by scientific advancements (publications) and innovation, but we advocate for a more sustainable, economical, and effective approach by minimizing the oscillations of boom and bust in funding and capacity for mosquito-borne viruses. Our goal should be to optimize the cost-effectiveness of budgetary spending by adopting resource allocation for biosecurity threats that maximize benefits while minimizing the total cost given anticipated expenditures incurred in the event of mosquito-borne viral outbreaks [49,50].
